# Comparative Pharmacokinetics of Hypaconitine after Oral Administration of Pure Hypaconitine, *Aconitum carmichaelii* Extract and Sini Decoction to Rats

**DOI:** 10.3390/molecules20011560

**Published:** 2015-01-16

**Authors:** Wen Zhang, Hai Zhang, Sen Sun, Feng-Feng Sun, Jun Chen, Liang Zhao, Guo-Qing Zhang

**Affiliations:** Department of Pharmacy, Eastern Hepatobiliary Surgery Hospital, Second Military Medical University, Shanghai 200438, China; E-Mails: nymph_zw_0212@sina.com (W.Z.); zhxdks2005@126.com (H.Z.); sunsen116@163.com (S.S.); sff10087@163.com (F.-F.S.); chenjuntcdj@126.com (J.C.); zhaoliangphar@hotmail.com (L.Z.)

**Keywords:** hypaconitine, *Aconitum carmichaelii*, Sini decoction, pharmacokinetics, LC-MS/MS

## Abstract

Hypaconitine (HC) is one of the main aconitum alkaloids in *Aconitum carmichaelii* (AC), which is considered to be effective on cardiovascular disease, although it also has high toxicity. Sini Decoction (SND), composed of *Aconitum carmichaelii*, *Glycyrrhiza uralensis* and *Zingiber officinale*, is a traditional Chinese multi-herbal formula for recuperating the depleted yang. The aim of this study was to compare the pharmacokinetics of HC in rat plasma after oral administration of HC, AC extract and SND, and investigate the effect of other two herbal ingredients on absorption, metabolism and elimination of HC. A sensitive and specific LC-MS/MS method was developed to determine HC in rat plasma. Eighteen male Sprague-Dawley rats were randomly assigned to three groups: HC, AC and SND group. Plasma concentrations of HC were determined at designated points after oral administration, and main pharmacokinetic parameters were estimated. It was found that there was obvious difference (*p* < 0.05) on the pharmacokinetic parameters among three groups. Compared with AC group, *T_max_*, *C_max_*,* k*, *AUC_(0-24)_* and *AUC_(0-∞)_* decreased in SND group, while *t_1/2_* and* MRT* had been lengthened, which indicated that the ingredients in other two herbs could influence the pharmacokinetic behavior of HC.

## 1. Introduction

*Aconitum carmichaelii* (AC) is widely used in traditional Chinese multi-herbal formulae for its analgesic, anti-inflammatory and cardiotonic actions [1,2,3]. The main bioactive ingredients in AC are highly toxic aconitum alkaloids [4], which are classified into three major groups: diester diterpene alkaloids (DDAs), monoester diterpene alkaloids (MDAs), and amine diterpenoid alkaloids (ADAs). The most toxic ingredients, including aconitine, mesaconitine, and hypaconitine [5], which can cause severe cardiotoxicity, neurotoxicity and cytotoxicity [6,7,8], are mainly derived from the DDAs. It is reported that the LD_50_ values of aconitine, mesaconitine, and hypaconitine after oral administration in mice are 1.8, 1.9, and 2.8 mg/kg [9,10], respectively. Due to the narrow therapeutic index of DDAs, it is very important to control the dose of DDAs in clinical applications.

To reduce the toxicity of AC, in traditional Chinese medicine it is used only after additional processing [11] and even more often in formulas containing other herbs [12,13]. Sini decoction (SND), composed of *Aconitum carmichaelii* (AC), *Glycyrrhiza uralensis* (GU) and *Zingiber officinale* (ZO), is a widely used traditional Chinese medicine for cardiovascular diseases [14,15]. GU and ZO could decrease the toxicity of AC through herbal interaction [16,17]. Because of pretreatment by boiling or some other methods, the DDAs could be hydrolyzed to MDAs [18]. Our previous study showed that the content of hypaconitine (HC) was much higher in the AC extract and SND than the other two DDAs. To control the toxicity of AC, the pharmacokinetic study of HC is important and necessary for us to understand.

Pharmacokinetic parameters could be different because of the processing process or herb interactions [19,20]. AC combined with GU or ZO could obviously affect the absorption and metabolism of DDAs [21,22], but there is little data showing the differences between the pharmacokinetic parameters of DDAs between the pure HC, AC extract and SND. Due to its high content in AC extract and SND, HC was chosen to study the pharmacokinetics after oral administration of pure HC to rats compared with AC extract and SND. The aim of this study was to develop a sensitive and specific LC-MS/MS assay for determination of HC in rat plasma, comparing the pharmacokinetics after oral administration of pure HC, AC extract and SND, and then further explore the effect of other herbal ingredients on the absorption and metabolism of HC.

## 2. Results and Discussion

### 2.1. Method Development

**Figure 1:**
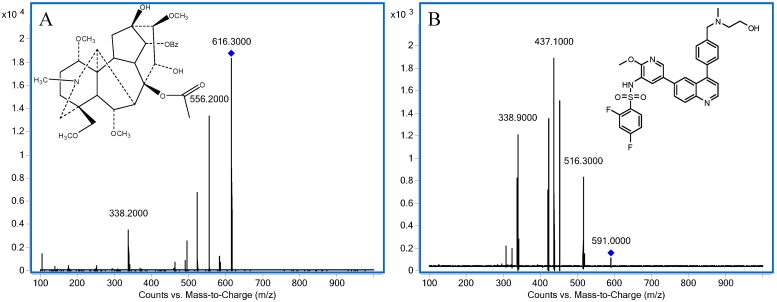
Representative MS/MS spectra and structures: (**A**) HC; (**B**) I.S.

The MS/MS parameters were optimized through an Agilent Automatic Optimizer to obtain the highest response using MRM pairs comprising the precursor and product ions, and quantification analysis was performed in positive ion mode. In the MRM mode, the optimized mass transition ion-pairs for quantification, including precursor and product ions, *m/z* 616.3→556.2 for HC and *m/z* 591.0→437.1 for I.S., respectively. Figure 1 displays the MS/MS spectra and structures of HC and I.S. The peaks obtained were separated well using the LC-ESI-MS/MS technique.

**Figure 2:**
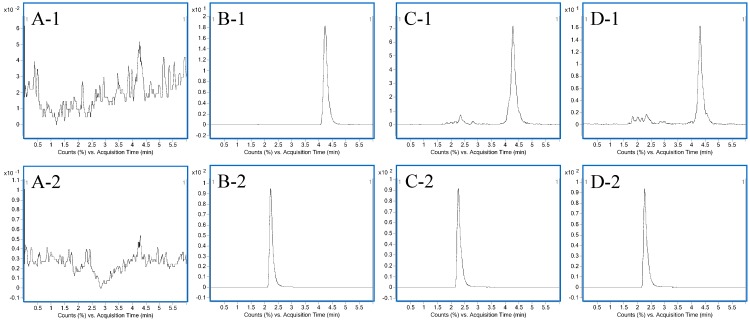
Representative chromatograms: (**A**) blank rat plasma; (**B**) rat plasma after oral administration of pure HC; (**C**) rat plasma after oral administration of AC extract; (**D**) rat plasma after oral administration of SND.1: HC; 2: I.S.

To obtain the appropriate retention time and responses, methanol, acetonitrile, water and formic acid were tested as mobile phases. Finally, acetonitrile-0.1% formic acid was found to lead to a higher response and lower background noise than the other mobile phases for all compounds tested. After optimization, the retention times of HC and I.S. were 4.232 and 2.218, respectively. Representative chromatograms of pure HC, AC extract and SND are shown in Figure 2.

For sample preparation, protein precipitation (PPT) and liquid-liquid extraction (LLE) were considered as work-up procedures. Because of the reported previously low recoveries of HC with liquid-liquid extraction [23], the protein precipitation method was used directly for its convenience and satisfactory recovery. Then, for the study of precipitation solvents, methanol and acetonitrile were investigated. The study showed acetonitrile could give satisfactory recovery and seemed to have less interference than the methanol extract and thus generate less matrix effects. The extraction efficiency using acetonitrile exceeded 90%, suggesting that it was an ideal precipitation agent.

### 2.2. Method Validation

The calibration curve of HC showed satisfactory linearity over the concentration range of 0.508–40.64 ng∙mL^−1^ while the regression equation was y = 1.348x − 1.123 and the correlation coefficient (r) was >0.99. Moreover, the LLOQ with a signal-to-noise (S/N) ratio >10 was 0.508 ng∙mL^−1^, whereas the LLOD with an S/N ratio >3 was 0.2032 ng∙mL^−1^, which was sensitive enough for the pharmacokinetic studies using rat plasma.

The intra- and inter-day precision of the method were assessed at three concentration levels of spiked analytes, and verified by determining the ratios of the peak areas to the internal standard with relative standard deviation (RSD) as listed in Table 1. The overall intra- and inter-day variation was less than 10%, indicating satisfactory precision of the instrumentation.

**Table 1:** Intra-day and inter-day precision and accuracy of HC in rat plasma (*n* = 5).

Analytes	Concentration (ng∙mL^−1^)	Intra-Day			Inter-Day		
		Measured (ng·mL^−1^)	RSD (%)	RE (%)	Measured (ng·mL^−1^)	RSD (%)	RE (%)
HC	1.016	1.055	2.697	3.839	1.074	3.945	5.709
	10.16	10.41	3.232	2.461	9.976	4.988	−1.811
	40.64	40.96	4.726	0.7874	40.76	2.517	0.2953

Table 2 shows that the extraction recoveries for HC and I.S. were greater than 80%, and no significant differences were found among the three concentrations. In addition, the matrix effect of analytes was all less than 10%, suggesting that the method was reliable and little matrix effect occurred.

**Table 2:** Matrix effect and extraction recoveries of HC and I.S. in rat plasma (*n* = 5).

Analytes	Concentration (ng∙mL^−1^)	Matrix Effects (%)	RSD (%)	Extraction Recovery (%)	RSD (%)
HC	1.016	93.23	3.572	89.43	4.329
	10.16	90.18	4.691	91.62	3.398
	40.64	91.56	3.866	92.79	3.854
I.S.	99.96	92.32	3.289	91.52	4.536

Analyte stability was assessed under various conditions. The results indicated that HC under three conditions (room temperature for 8 h, −40 °C for 30 days, freeze-thraw for 3 times) were all stable in plasma and there was no significant degradation (RE < 15%) and all the results are displayed in Table 3.

**Table 3:** Stability of HC in rat plasma (*n* = 5).

Analytes	Concentration (ng·mL^−1^)	Frozen at −40 °C for 30 Days		Room Temperature for 8 h		Freeze-Thaw Cycles	
		Measured (ng·mL^−1^)	RE (%)	Measured (ng·mL^−1^)	RE (%)	Measured (ng·mL^−1^)	RE (%)
HC	1.016	1.056	3.94	1.007	−0.89	0.998	−1.77
	10.16	10.37	2.07	10.58	4.13	9.89	−2.66
	40.64	41.23	1.45	40.32	−0.79	41.98	3.30

### 2.3. Pharmacokinetic Study

**Figure 3:**
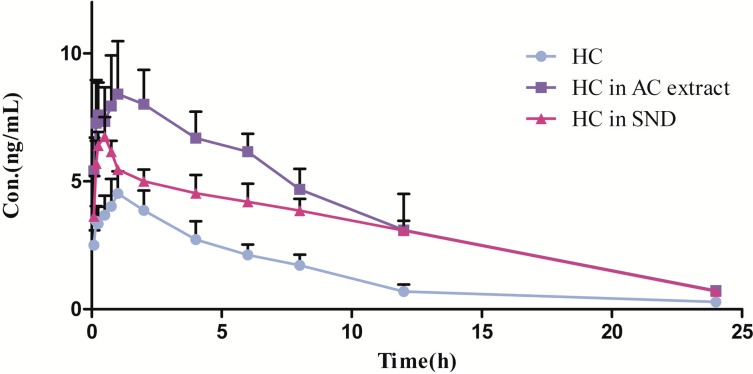
Representative mean concentration-time curve of HC after oral administration of pure HC, AC extract and SND (*n* = 6).

The analytical procedures, described in Section 3.2 were applied to quantify HC in rat plasma samples obtained from 18 male SD rats, of which six were orally administered pure HC, six were orally administered AC extract, and the other six were orally administered SND. The mean concentration-time curve of HC in three groups is presented in Figure 3. The pharmacokinetic parameters of HC in male SD rats following oral administration of pure HC, AC extract and SND were calculated by DAS 3.0 with the non-compartmental model. The fitted pharmacokinetic parameters are shown in Table 4.

The area under the concentration-time curve from 0 h to the last experimental point 24 h (*AUC_(0−24)_*) was estimated by the linear trapezoidal rule. End half-life (*t_1/2_*) was calculated as *t_1/2_* = 0.693/*k*, and *k* was determined by linear regression of the logarithmical plasma concentration* versus* time for the last 4 data points in the concentration-time curve. *C_max_* and *T_max_* values were obtained directly from the observed concentration-time curves. One-way ANOVA and Newman-Keuls test was used for comparison (*p* < 0.05). All results were expressed as arithmetic mean ± standard deviation (S.D.).

As shown in Table 4, the plasma concentrations of HC increased rapidly after oral administration to rats, and the *T_max_* in the HC, AC extract and SND groups was 1.125 ± 0.440, 1.083 ± 0.466, 0.4583 ± 0.1882 h, respectively. The *T_max_* in the SND group was much lower than that in the other two groups, indicating the faster absorption rate in this group. The *C_max_* in the HC group, 4.751 ± 0.930 ng·mL^−^^1^, was much lower than that in the AC extract and SND groups. A similar trend could be observed in *AUC_(0-24)_* and *AUC_(0-__∞__)_* among the HC, AC extract and SND groups. The increasing *C_max_*, *AUC_(0-24)_* and *AUC_(0-__∞__)_* showed the better absorption of HC in the AC extract and SND groups. It may be inferred that some ingredients in the AC extract could enhance the absorption of HC. There was no significant difference in the *k* and *t_1/2_* parameters between the HC and AC extract groups, while there was a significant difference between the AC extract and SND groups, indicating the elimination of HC was lengthened in SND. This might be due to the other ingredients in SND affecting the elimination of HC.

**Table 4:** Pharmacokinetic parameters of HC after oral administration of pure HC, AC extract and SND (*n* = 6).

Parameters	Unit	Pure HC	AC Extract	SND
*T_max_*	h	1.125 ± 0.4402	1.083 ± 0.4655	0.4583 ± 0.1882 *^▲^
*C_max_*	ng·mL^−1^	4.751 ± 0.9300	9.758 ± 0.6320 *	6.880 ± 0.6091 *^▲^
*k*	h^−1^	0.1605 ± 0.0493	0.1442 ± 0.0345	0.1020 ± 0.0107 *^▲^
*t_1/2_*	h	4.633 ± 1.246	5.015 ± 1.046	6.855 ± 0.732 *^▲^
*AUC_(0-24)_*	ng·mL^−1^·h	33.72 ± 5.311	92.28 ± 12.39 *	73.80 ± 1.731 *^▲^
*AUC_(0-__∞)_*	ng·mL^−1^·h	34.90 ± 5.270	96.44 ± 14.26 *	80.79 ± 1.037 *^▲^
*CL*	L·h^−1^·kg^−1^	5.842 ± 0.8867	2.113 ± 0.3209 *	2.476 ± 0.0318 *^▲^
*MRT*	h	7.125 ± 0.5493	8.265 ± 1.304 *	10.32 ± 0.6720 *^▲^

Notes: * *p < 0.05* compared with HC group; ^▲^ *p < 0.05* compared with AC extract group.

Compared with the AC extract, HC was absorbed more rapidly after oral administration of SND. In spite of the fast absorption rate, the parameters of *C_max_*, *AUC_(0-24)_* and *AUC_(0-∞)_* were sharply lower, with *C_max_* in 6.880 ± 0.6091 ng·mL^−1^, *AUC_(0-24)_* in 73.80 ± 1.731 ng·mL^−1^·h, *AUC_(0-∞)_* in 80.79 ± 1.037 ng·mL^−1^·h, which indicated some ingredients in GU and ZO affect the absorption of HC and inhibit absorption of HC in SND. There were significant differences in the parameters of *k*, *t_1/2_* and *MRT* between the AC extract and SND groups, showing that the elimination of HC was lengthened in SND. All the above proved that there probably existed herbal interactions in the compound herbal formula. Many case reports have proved that GU and ZO could help reduce the toxicity and increase the efficacy of AC. The pharmacokinetic parameters of HC in the present study revealed that AC in SND could maintain the concentration of HC in a relatively moderate range and ensure its efficacy through combining with GU and ZO.

## 3. Experimental Section

### 3.1. Materials and Reagents

The HC reference standard (purity > 98%) was obtained from China’s National Institute for the Control of Pharmaceutical and Biological Products (Beijing, China), A26 (purity > 98%) as an internal standard was synthesized by our laboratory. Acetonitrile and formic acid of HPLC grade were from Burdick & Jackson (Muskegon, MI, USA). Ultrapure water was prepared by a Milli-Q System (Millipore, Bedford, MA, USA). All other reagents were of analytic grade. *Aconitum carmichaelii* (AC), *Glycyrrhiza uralensis* (GU) and *Zingiber officinale* (ZO) were purchased from the Shanghai Leiyunshang Pharmaceutical Limited Company (Shanghai, China) and authenticated by Professor Lianna Sun from the Department of Pharmacognosy, Second Military Medical University (Shanghai, China).

### 3.2. Instrumentation and Conditions

The HPLC-MS/MS system consisted of an Agilent 1260 RRLC and an Agilent 6410 Triple Quadruple mass spectrometer (Agilent, Santa Clara, CA, USA). The quantification was performed using multiple-reaction monitoring (MRM) with electrospray ionization (ESI) operated in the positive-ion mode. The separation was carried out on Waters Xbridge C_18_ column (100 × 3.0 mm, 3.0 μm) (Waters, Milford, MA, USA) with an Agilent-C_18_ (12.5 × 4.6 mm, 5 µm guard column). The mobile phase consisted of A (H_2_O: HCOOH, 100:0.1, v/v) and B (acetonitrile) (65:35, v/v). The column temperature was 25 °C. The total analysis time was 6 min. The injection volume of 5 μL and the flow rate was 0.8 mL·min^−1^, which was introduced into the mass spectrometer by a post-column splitting ratio of 1:1 with a three-way joint.

The MS instrumental settings were as follows: capillary voltage −3.5 kV, Nozzle voltage 500 V, nebulizer gas pressure 45 psig, drying gas flow rate 11 L·min^−1^, gas temperature 350 °C, sheath gas temperature 400 °C and sheath gas flow at 11 L·min^−1^. The Agilent MassHunter Worksatation Data Acquisition software was used for equipment control and data acquisition, and Agilent Qualitative Analysis software was used for data analysis.

### 3.3. Preparation of AC Extract and SND

AC, GU and ZO were crushed to pieces before using. AC pieces (60 g) and SND (AC 60 g, GU 60 g, ZO 40 g) were decocted twice in boiling water (1:6, w/v) for 2 h and then another 0.5 h, and finally the extracted solution was concentrated to 50 mL, 95% ethanol (150 mL) was added for 24 h, the mixture was centrifuged at 2000 rpm for 10 min, and the supernatant was concentrated to 24 mL to obtain the AC extract and SND. The HC content in the AC extract and SND were measured quantitatively by HPLC-DAD. HC was separated on a Waters Xbridge C_18_ column (100 × 3.0 mm, 3.0 μm) and eluted with the same LC conditions used at the UV detection wavelength of 235 nm. SND and AC extract were filtered with a 0.45 μm Millipore filter before injection into the LC system. Compared with the areas of hypaconitine standard solution, the contents of HC were calculated as 21.03 μg·mL^−1^ in AC extract and 20.40 μg·mL^−1^ in SND.

### 3.4. Preparation of Calibration Standards, Quality Control and Internal Standard

Stock solution of HC was prepared by dissolving accurately weighed standard compound in acetonitrile at a concentration of 508.0 μg·mL^−1^. The internal standard A26 was dissolved with acetonitrile to 999.6 μg·mL^−1^ as the internal standard stock solution. HC standard working solutions were prepared by further combining the aliquots of each primary stock solution and diluting to the concentrations of 40.64, 20.32, 10.16, 5.08, 2.032, 1.016, 0.508 ng·mL^−^^1^ and the internal standard was diluted to 99.96 ng·mL^−^^1^ before use. The assay standard samples were prepared by spiking 100 μL blank rat plasma with 20 μL of the working standard solutions at different concentrations. The quality control samples at 40.64, 10.16, 1.016 ng·mL^−^^1^ of HC were independently prepared in the same manner. All the solutions were kept at 4 °C before analysis. The assay standard samples and quality control samples were analyzed in every analysis batch.

### 3.5. Sample Preparation

To 100 μL aliquot of a plasma sample, acetonitrile (20 μL) and internal standard solution (180 μL, 99.96 ng·mL^−1^) were added in a 1.5 mL polypropylene tube. After vortex-mixing for 30 s and centrifuging at 12,000 rpm for 10 min, the mixture was transferred into the injection vials, and a 5 μL aliquot of the supernatant was injected into the LC-MS/MS system for analysis.

### 3.6. Validation of LC-MS/MS Method

For calibration curve generation, six concentrations of calibration standards were prepared by diluting the working solution. The serial solutions (20 μL of each concentration) were added into 100 μL of blank plasma with 180 μL of internal standard solution and determined as described above. The calibration curve for HC was constructed based on the peak area ratios of the analyte to I.S. against plasma concentrations. Method selectivity was assessed by comparing chromatograms of six individual blank rat plasma samples with those obtained by spiking analytes and I.S. into the blank plasma sample.

The lower limit of detection (LLOD) and lower limit of quantitation (LLOQ) were determined as the concentration of the analytes with a signal-to-noise ratio at 3 and 10 in the blank plasma, respectively. The extraction recovery was determined by comparing the peak areas of QC samples at the concentrations of 40.64, 10.16, 1.016 ng·mL^−1^ with those of the post-extracted blank plasma spiked with analytes at the same concentration, and this was replicated five times. The matrix effect was evaluated by comparing the solution spiked with the blank processed matrix with those of the corresponding standard solution at three different QC concentrations and this was replicated five times. The extraction recovery and matrix effect of I.S. was determined in the same way.

Samples at three different concentrations were determined five times in the same day to measure the intra-day accuracy and over three consecutive days to measure the inter-day accuracy. Each concentration, five replicates were prepared while the content was calculated using a calibration curve on the same testing day. For measurement of sample stability, three sets of samples under different storage conditions were prepared for analysis. These samples were stored at room temperature for 8 h, at −40 °C for 30 days and at −40 °C for three freeze-thaw cycles.

### 3.7. Study in Vivo

This animal experimental protocol was carried out according to the Guidelines for the Care and Use of Laboratory Animals, and was approved by the Animal Ethics Committee of the Second Military Medical University. Male Sprague-Dawley (SD) rats (*n* = 18) weighing 250–280 g were supplied by Sino-British Sippr/BK Lab Animal Ltd. (Shanghai, China). The rats were housed under standard conditions of at 22 ± 2 °C and 50% ± 10% relative humidity with water and food (laboratory rodent chow, Shanghai, China) allowed *ad libitum*. The animals were acclimated to the facilities for at least five days, and then fasted overnight with free access to water prior to each experiment. SD rats were randomly divided into three equal groups: HC, AC extract and SND groups. HC was dissolved in water at the concentration of 1 mg·mL^−^^1^, and diluted to 20 μg·mL^−^^1^. HC water solution, AC extract and SND were orally administrated at the dosage of 200 μg·kg^−1^ for each group. Blood samples (0.3 mL) were collected from the orbital plexus of the eyes at the designated time points (0.083, 0.167, 0.25, 0.5, 0.75, 1, 2, 4, 6, 8, 12 and 24 h) after drug administration. All the blood samples were collected in the 1.5 mL polypropylene tubes which were distributed with heparin. After centrifuge at 4000 rpm for 10 min, plasma samples were obtained and frozen at −40 °C until analysis.

### 3.8. Pharmacokinetic Study

The pharmacokinetic parameters were calculated using DAS version 3.0 (BioGuider Co., Shanghai, China). The following parameters were calculated through the non-compartmental model: relating to area under the plasma concentration (*AUC*)-time curve, maximum concentration (*C_max_*), the time for maximum concentration (*T_max_*), elimination rate constant (*k*), half-life (*t_1/2_*), clearance (*CL*) and mean residence time (*MRT*). Comparisons among the three groups were performed using one-way ANOVA and Newman-Keuls test. The value of *p <* 0.05 was considered statistically significant.

## 4. Conclusions

In the present study, an LC-MS/MS combined with a direct precipitation method was developed to quantify the content of hypaconitine in rat plasma. Then, the method was successfully applied to a pharmacokinetic study after oral administration of pure HC, AC extract and SND to rats. The study results showed that there were significant differences among pharmacokinetic parameters of HC after oral administration of pure HC, AC extract and SND, indicating that other ingredients in the process of processing or boiling could result in pharmacokinetic differences of HC. The results might help explain the action mechanism of traditional Chinese multi-herbal formula.
